# Scintillations from the Pellagra Symposium

**Published:** 1915-12

**Authors:** 


### Scintillations From the Pellagra Symposium.

The treatment of pellagra by diet as announced recently by Dr. Joseph H. Goldberger of the United States Public Health Service was not accepted as a positive cure in discussion among physicians at the pellagra symposium of the Southern Medical Association's annual meeting. The differences of opinion expressed were sharp. There were no criticisms of Dr. Goldberger's work, but there was considerable unanimity of opinion that news reports of his work appearing in the "lay press" have been misleading.

Dr. Allan W. Freeman of the United States Public Health Service of Washington read to the symposium a copy of the diet which Dr. Goldberger found had a tendency to produce pellagra. Dr. Freeman read it, and pointed out that it was almost the identical diet of every person of poor means in the South, appealed to the physicians to accept for their own use and experiment the treatment Dr. Goldberger had demonstrated.

"Dr. Goldberger," he said, "does not claim to prove that diet is the only cause for pellagra. But he does claim that through diet he has prevented pellagra, and that through diet he has produced it."

Dr. Stewart L. Roberts, of Atlanta, said that as pellagra is a wasting disease, a diet, including such foods as peas, beans and fresh meats, which were among the articles recommended by Dr. Goldberger, would tend toward relief for the reason that they are rich in the elements necessary to restore wastage.

"However," he said, "we should have much more evidence than that yet submitted to prove Dr. Goldberger's theory of diet."

Dr. Roberts added that he did not believe it possible to say a pellagra patient had recovered until the passage of at least three years, during which not a single symptom of the disease appeared. He thought that when the cause of pellagra is discovered it will be either a parasite or a poison.

Dr. W. L. Allison, of Fort Worth, Chairman of the State at Large Committee on Pellagra, offered an exceptionally interesting paper on State conditions. That the disease is on the increase Dr. Allison admitted, yet he challenged the contention that it exists merely among the poorer classes.

He said that there are many reasons to believe that pellagra came to America from Italy, where it has thrived for 200 years and where its treatment has been as uncertain as in America. There was no proof, he said, that pellagra existed in the United States prior to 1906. Since 1907, he said, the disease has spread rapidly throughout the South until now each State numbers its pellagra cases by the tens of thousands. He expressed the belief that the disease is not contagious because doctors and nurses attending pellagra patients have not become infected. The matter of diet and rundown constitutions, he said, appears to be important factors, although he said this is not always the case, as instances have been found where no fault could be attached to the diet of pellagra patients. The disease, he said, was more prevalent where there are unscreened privies and where water carriage sanitary sewers are not provided. He also expressed the opinion that under favorable conditions 80 per cent of the cases get well.

"Pellagra in Texas" was the subject dealt with in a paper by Dr. K. H. Beall, of Fort Worth. He criticised the State government for apparent apathy in checking the ravages of the disease. Last year he said more than five hundred victims had been claimed by pellagra and several hundred additional persons had been made invalids by it. He said it had been eight years since the first case was reported in Texas, and displayed some interesting charts showing counties in which deaths had occurred from pellagra. Negro girls, Dr. Beall declared, were more prone to pellagra than white children.

"The State of Texas," he said, "has no right to sit complacently by while this enemy to the Commonwealth slays its people. No one knows but that pellagra has found a more fertile field in Texas than in Italy."

The first paper on the symposium was read by Dr. H. Leslie Moore, of Dallas, as the Chairman of the Committee on Prevention and Treatment of Pellagra, designated by the Dallas Medical and Surgical Society last August. Dr. Moore commended the recent experiments with pellagra at Jackson, Tenn., and expressed the belief that Dr. Joseph Goldberger's theory is the most plausible and the only one that comes anywhere near being substantiated. He declared that few cases occur among classes other than the poor. Dr. Goldberger had observed, he said, in certain orphanages in Mississippi a large number of children between the ages of 6 and 12 years were affected. Investigation developed that these children had been fed very little lean meat or other animal protein food, that they had been fed mostly biscuits, meal, syrup and very little of beans, peas, etc. Similar conditions found at different orphanages had led him to believe that the dietary conditions constitute a potent if not elementary factor in dealing with pellagra. Dr. Goldberger, he said, recommends the proper dieting among pellagra patients in the late winter and early spring months and the individual ownership of more milch cows and increased milk production for home consumption. He also emphasizes poultry and egg production for home consumption.

He also advised a movement in economic conditions which would result in increasing wages so that the poorer classes where pellagra is prevalent may provide themselves with the proper food.

Dr. W. A. Dearman, of Long Beach, Miss., read a paper on "The Further Consideration of the Etiology of Pellagra With Reference to Amebic Invasion." He cited the case of the State Hospital at Peoria, where, during 1909, pellagra claimed a mortality rate of about 50 per cent of a large number affected. The disease first made its appearance in 1907 and involved more than 8 per cent of the inmates of the institution. This institution, he said, is provided with an extra water carriage system of sewerage.

Dr. O. M. Marchman, of Dallas, said whatever the final proof on diet treatment may show, even if the treatment fails, it should start study with renewed vigor and hope of soon getting pellagra under control. He said there are probably between 35,000 and 50,000 pellagra cases in Texas. Dr. K. H. Beall, of Fort Worth, said that in 1907 the first pellagra death was reported in Texas, and that last year 500 deaths were reported.

